# Formulation of a Mixture of Plant Extracts for Attenuating Postprandial Glycemia and Diet-Induced Disorders in Rats

**DOI:** 10.3390/molecules24203669

**Published:** 2019-10-11

**Authors:** Adam Jurgoński, Katarzyna Billing-Marczak, Jerzy Juśkiewicz, Marcin Krotkiewski

**Affiliations:** 1 Division of Food Science, Institute of Animal Reproduction and Food Research, Polish Academy of Sciences, 10-748 Olsztyn, Poland; j.juskiewicz@pan.olsztyn.pl; 2 Department of Research and Development at MarMar Investment Company, 10-195 Warsaw, Poland; 3 Department of Neurological Rehabilitation, Gothenburg University Hospital, SE-405 30 Gothenburg, Sweden

**Keywords:** α-glucosidase inhibitors, carbohydrate digestion, glucose absorption, impaired glucose tolerance, plant extracts

## Abstract

The aim of this study was to design a mixture consisting of plant-derived preparations containing inhibitors of carbohydrate digestion and/or glucose absorption that could lower postprandial glycemia and attenuate dietary-induced disorders. The following standardized preparations were tested: white mulberry leaf extract, green coffee bean extract, white kidney bean extract, pomelo fruit extract, bitter melon fruit extract, and purified l-arabinose. The study design was composed of oral sucrose and starch tolerance tests in Wistar rats preceded by a single ingestion of the preparations or their mixtures. Then, a 20 week-long experiment was conducted on rats that were fed a high-fat diet and supplemented with the most effective mixture. Based on the results of the oral sucrose and starch tolerance tests, the mulberry leaf extract, l-arabinose, kidney bean extract, and coffee bean extract were selected for composing three mixtures. The most effective inhibition of postprandial glycemia in the oral tolerance tests was observed after the ingestion of a mixture of mulberry leaf, kidney bean, and coffee bean extract. The glucose-lowering effect of the mixture and its effective dosage was confirmed in the feeding experiment.

## 1. Introduction

An excessive intake of refined carbohydrates has been linked to the epidemic increase of obesity and type 2 diabetes worldwide [1,2]. Impaired glucose tolerance and insulin resistance are key disorders of glucose metabolism that lead to the development of metabolic syndrome and type 2 diabetes. Numerous studies have also shown that increased blood glucose levels are an independent risk factor for cardiovascular comorbidities [3,4]. 

One of the approaches for lowering postprandial glycemia is the inhibition of carbohydrate digestion or absorption in the gastrointestinal tract. Carbohydrate digestion and absorption is a multistage process occurring in different parts of the gastrointestinal tract, and it starts in the oral cavity by the enzyme ptyalin, which is a salivary amylase. However, the crucial mechanisms of carbohydrate digestion and absorption occur in the small intestine, where pancreatic α-amylase, responsible for polysaccharide digestion, mainly starch, is secreted into the duodenum [5]. Then, oligosaccharides and disaccharides are digested by intestinal amylase and mucosal disaccharidases, among which maltase, sucrase, isomaltase, and lactase are the most important. Products of the digestion process are simple sugars, mainly glucose, that are actively absorbed into the enterocytes by sodium-dependent glucose cotransporter 1 (SGLT1) or, to a lesser extent, by facilitated diffusion through glucose transporter 2 (GLUT2).

There are few antidiabetic drugs available on the market that lower postprandial glycemia through inhibiting intestinal α-glucosidase activity, such as acarbose or miglitol. However, these drugs are not recommended for healthy people, and many adverse effects after their intake may occur, such as flatulence, abdominal distension, stomach rumbling, and diarrhea [6]. Thus, there is a need for developing dietary supplements, preferably consisting of natural compounds, which could be useful for individuals in any health stage who want to prevent high postprandial glycemia and, in the long term, obesity and type 2 diabetes. Many plant-derived compounds can affect carbohydrate digestion or absorption in the gastrointestinal tract. One of them is l-arabinose, a pentose broadly present in plants as a structural component of polysaccharides, such as hemicelluloses, pectins, and gums, of which it is commonly isolated [7]. Animal studies and clinical trials have shown that l-arabinose is able to effectively reduce postprandial blood glucose levels, most likely through the uncompetitive inhibition of sucrase and maltase [8,9]. Another intestinal disaccharidase inhibitor is iminosugars found in mulberry leaves, mainly 1-deoxynojirimycin, which are able to inhibit maltase, isomaltase, and sucrase activity [10]. A natural occurrence of α-amylase inhibitors has also been recognized in the literature. One of them is phaseolamin, a proteinaceous compound isolated from kidney beans of proven glucose- and body weight-lowering activity [11,12]. Another α-amylase inhibitor can be found in large quantities in coffee beans, which is chlorogenic acid, a phenolic acid that is able to reduce plasma glucose peaks [13,14]. This and other caffeoylquinic acids have also been reported as strong inhibitors of mucosal disaccharidase activities [15,16]. Furthermore, an example of compounds that can affect glucose uptake is naringenin, a polyphenol belonging to flavanones, which is present in citrus fruits, such as grapefruits or pomelo fruits. This polyphenol can inhibit intestinal SGLT1 and thus is also considered a potential glycemia-lowering agent [17]. The presence of glucose uptake inhibitors has also been reported in bitter melon fruits; however, the exact compound responsible for this effect has not yet been identified. Nevertheless, extracts from bitter melon fruits have been frequently recognized as having hypoglycemic activity, which is also partly due to the presence of insulin-like peptides in this plant [18].

The aim of this study was to design a mixture consisting of different plant extracts and containing inhibitors of carbohydrate digestion and/or glucose absorption that could lower postprandial glycemia and attenuates dietary-induced disorders in rats. Based on the available literature, we chose to research l-arabinose and extracts from the following plants: white mulberry, white kidney beans, coffee beans, bitter melon, and pomelo. Considering that different plant-derived compounds have their own mechanisms of glycemia-lowering action and other beneficial effects, we hypothesized that their mixtures would exert a superior effect on postprandial glycemia than each of the preparations separately, whereas a mixture exhibiting optimal glycemia-lowering action can additionally attenuate dietary-induced disorders in rats.

## 2. Results

The total area under the blood glucose curve (AUC) for the oral sucrose and starch tolerance tests (SuTT and StTT, respectively) in rats gavaged with acarbose, arabinose, or the extracts is shown in Table 1. For both the SuTT and StTT, the lowest AUC values were noted in the acarbose group, and they were significantly lower than in the other groups. In the SuTT, the AUC values were also significantly reduced in the coffee bean extract (CBE), mulberry leaf extract (MLE), kidney bean extract (KBE), and pomelo fruit extract (PFE) group compared to that of the control group, and the mulberry leaf extract tended to be the most efficient of the tested extracts. The AUC values for the StTT were significantly decreased in the arabinose, MLE, and PFE groups compared to that of the control group, and the mulberry leaf extract, as well as arabinose, tended to be the most efficient. Based on these results and based on the glycemic data for each preparation, the mulberry leaf extract, arabinose, kidney bean extract, and coffee bean extract were selected for composing mixtures for further tests. 

**Figure 1:**
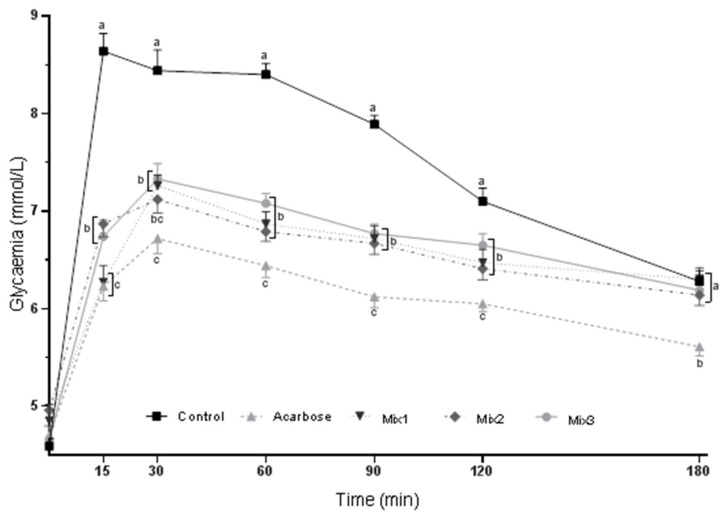
Blood glucose curves for the oral sucrose tolerance test (SuTT) in rats gavaged with acarbose or the composed mixtures. Values are expressed as the mean ± SEM (*n* = 12). Mean values with differing letters within a time period (a, b, c) are significantly different at *p* < 0.05 in a *post hoc* test. BW, body weight. Composition and doses (mg/kg BW): Mix1—mulberry leaf extract (120 mg) + kidney bean extract (100 mg) + coffee bean extract (20 mg); Mix2—mulberry leaf extract (120 mg) + coffee bean extract (20 mg) + arabinose (25 mg); Mix3—mulberry leaf extract (120 mg) + kidney bean extract (100 mg) + coffee bean extract (20 mg) + arabinose (25 mg).

**Figure 2:**
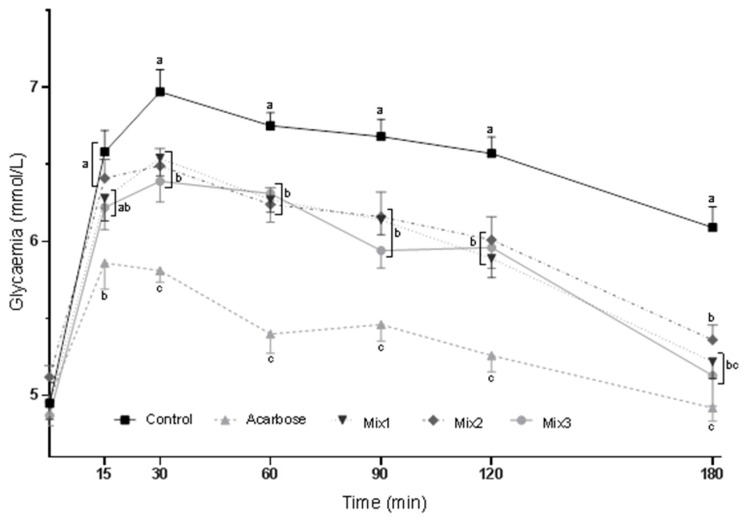
Blood glucose curves for the oral starch tolerance test (StTT) in rats gavaged with acarbose or composed mixtures. Values are expressed as the mean ± SEM (*n* = 12). Mean values with differing letters within a time period (a, b, c) are significantly different at *p* < 0.05 in a *post hoc* test. BW, body weight. Composition and doses (mg/kg BW): Mix1—mulberry leaf extract (120 mg) + kidney bean extract (100 mg) + coffee bean extract (20 mg); Mix2—mulberry leaf extract (120 mg) + coffee bean extract (20 mg) + arabinose (25 mg); Mix3—mulberry leaf extract (120 mg) + kidney bean extract (100 mg) + coffee bean extract (20 mg) + arabinose (25 mg).

The blood glucose curves for the SuTT in rats gavaged with acarbose or composed mixtures are depicted in Figure 1. After 15 min of sucrose gavage, all three mixtures significantly decreased the glycemia of rats compared with that of the control group, but only mixture 1 lowered it to a level comparable with that of acarbose, whose inhibitory activity was the most efficient at each time point of the test (group Mix1, Figure 1). After 30, 60, 90, and 120 min of sucrose ingestion, a similar glycemia decrease was observed in all three mixture groups compared to that of the control group. At the end of the SuTT, glycemia was comparable among all mixture groups and the control group (180 min of the test). Blood glucose curves for the StTT in rats gavaged with acarbose or formulated mixtures are depicted in Figure 2. After 15 min of starch ingestion, mixtures 1 and 3 slightly decreased glycemia, but to a level comparable with that of the acarbose group. After 30, 60, 90, and 120 min of the starch gavage, a similar glycemia decrease was noted in all three mixture groups compared to that of the control group; however, the lowest glycemia was still found in the acarbose group. At the end of the StTT, glycemia was significantly reduced by all three mixtures; however, in the Mix1 and Mix2 groups, it was slightly more reduced and comparable with that of the acarbose group (180 min of the test) than in the Mix3 group. Moreover, the AUC values for the SuTT and StTT were similar among the mixture groups and were significantly lower than that of the control group; however, the lowest AUC values were still noted in the acarbose group (Table 2). Based on these results, mixture 1, composed of the mulberry leaf, kidney bean, and coffee bean extracts, was chosen for the feeding experiment. 

The effects of mixture 1 on the diet intake, body weight, and body composition of rats fed a high-fat diet on the proper stage of the experiment are shown in Table 3. The initial body weight (BW) and body fat percentage of rats only increased (*p* > 0.05), whereas the initial body lean percentage significantly decreased in the high-fat (HF) and HF+Mix1 groups compared to that of the control group. This was the result of the preliminary stage, in which rats were fed a commercial high-fat diet for 10 weeks. After 10 weeks of proper feeding with semi-purified diets, the diet intake was reduced, whereas the BW increased in both HF groups compared to that of the control group. Moreover, the final body fat percentage increased, whereas the final body lean percentage decreased in both HF groups compared to those of the control group. Nevertheless, the BW and body fat gains were significantly decreased in the HF+Mix1 group compared to those of the HF group, but their lowest values were still observed in the control group. Rats from the HF groups also had negative body lean gain compared with that of the control. 

The effects of mixture 1 on the plasma markers of glucose and lipid metabolism in rats fed a high-fat diet for 10 weeks are shown in Table 4. The glucose concentration was significantly lower in the HF+Mix1 group than in the HF group, whereas the values did not differ from that of the control group. The insulin concentration was similar in all groups. The non-HDL cholesterol concentration significantly increased in the HF group compared to that of the control and HF+Mix1 groups.

## 3. Discussion

The primary aim of this study was to design a mixture consisting of different plant extracts containing inhibitors of carbohydrate digestion and/or glucose absorption that could lower postprandial glycemia in rats. Extracts containing inhibitors of mucosal disaccharidase activity (arabinose, mulberry leaf extract, and coffee bean extract), α-amylase activity (coffee bean extract and kidney bean extract), glucose absorption (pomelo fruit extract and bitter melon fruit extract), and other activities (bitter melon fruit extract) were chosen for the tests [8,9,10,11,12,13,14,15,16,17,18]. Acarbose, which is a well-recognized inhibitor of intestinal α-glucosidase activity, was chosen as a positive control, and in both tolerance tests, this medicine most effectively reduced postprandial glycemia in rats. In addition to acarbose, an effective reduction of postprandial glycemia in the SuTT was observed after the ingestion of mulberry leaf extract, coffee bean extract, kidney bean extract, and pomelo fruit extract, whereas in the StTT, an effective reduction of postprandial glycemia was observed after the ingestion of arabinose, mulberry leaf extract, and pomelo fruit extract. Generally, our results suggest that mucosal disaccharidase inhibitors, due to their capability to disrupt the final stage of carbohydrate digestion, may play a more crucial role in reducing postprandial glycemia than inhibitors of α-amylase activity or glucose absorption. First, we confirmed the glucose-lowering effect of mulberry leaf extract, which is extensively reported in the literature as containing iminosugars that are able to inhibit mucosal disaccharidase activities [10,19]. However, we also obtained some results that are more or less contradictory to the existing literature data. For instance, the suppression of postprandial glycemia by arabinose was much more associated with starch than sucrose ingestion, and those results have been extensively discussed in our preliminary report [9]. Moreover, the chlorogenic acid-rich extract from coffee beans was able to decrease postprandial glycemia in the SuTT, but not in the StTT. This finding confirms an inhibitory activity of chlorogenic acid and other caffeoylquinic acids towards mucosal sucrase [16,20] but does not support its intestinal α-amylase inhibitory activity [14]. Moreover, we used a kidney bean extract with a 4000 units/g amylase inhibitory activity that was determined in vitro, but in our in vivo tests, the activity was not confirmed. This finding is in accordance with those reported by other scientists who performed in vivo studies, including clinical trials, and failed to observe any measurable inhibition of starch hydrolysis or significant weight loss, indicating either the existence of an alternate route capable of degrading starch or insufficient amounts of inhibitors reaching the substrate [19]. Surprisingly, the results of the present study suggest some potential inhibition of sucrase activity by the kidney bean extract; however, this needs further research. In the present study, a lack of a glucose-lowering effect of the bitter melon fruit extract and a relatively low effective potential of the pomelo fruit extract in this matter are also noteworthy. Although some effect of the pomelo fruit extract was noted in the SuTT, the glycemic response was significantly lower only at the end of the test compared to that of the control (180 min, S1. Appendix). Taking all of the aforementioned results into consideration, we decided to choose the following preparations for mixture formulations: arabinose, mulberry leaf extract, coffee bean extract, and kidney bean extract. 

Three mixtures were composed of the tested preparations and used for the tolerance tests in stage 2 of the study: mixture 1 (mulberry leaf extract + kidney bean extract + coffee bean extract), mixture 2 (mulberry leaf extract + coffee bean extract + arabinose), and mixture 3 (mulberry leaf extract + kidney bean extract + coffee bean extract + arabinose). All preparations were applied in the same doses as previously described, and the mulberry leaf extract was the basis of each mixture, as it was found to have the most potent glucose-lowering activity in both tests. According to the AUC, the most effective glucose reduction in the tolerance tests was observed after acarbose gavage (Table 2). The mixtures were also effective, and they were able to reduce postprandial glycemia in a similar manner in both tests according to the AUC. Nevertheless, after 15 min of the SuTT, mixture 1 was as effective as acarbose, which was not the case for the other mixtures (Figure 1). Additionally, at the beginning and end of the StTT (15 min and 180 min, respectively), mixture 1 and mixture 3 were able to decrease glycemia to a level comparable with that of the acarbose group (Figure 2). These results show that there is a possibility to formulate a mixture of plant extracts that is partly as effective as acarbose. 

As a result of the tolerance tests conducted in stage 2, mixture 1 consisting of mulberry leaf, kidney bean, and coffee bean extracts in the mass percentages of 50, 42, and 8, respectively, was selected for the feeding experiment, the specific aim of which was to evaluate whether this formulation is able to attenuate disorders induced by an HF diet in rats. An energy-dense diet rich in fat is often used to induce obesity and disorders related to glucose and lipid metabolism in laboratory animals [21]. For this purpose, a 10 week-long preliminary stage with HF dietary feeding and a 10 week-long proper stage with an HF diet plus mixture 1 feeding was conducted. The preliminary stage was used to make the rats more prone to obesity, and at the beginning of the proper stage, they had a significantly decreased body lean percentage; however, the body weight and fat percentage only increased (initial body parameters, Table 3). The diet intake after 10 weeks of the proper stage of HF feeding significantly decreased, and mixture 1 did not affect this outcome. This phenomenon was a consequence of the increased energy density of both HF diets, to which rats had to partly adapt themselves by reducing dietary intake. Nevertheless, the proper HF feeding was still able to stimulate body weight and fat gain, leading to an experimental form of obesity, whereas mixture 1 was able to significantly reduce these indices but not to the levels of the indices in the control group. These results confirm previous knowledge on the anti-obesity effects of compounds found in white mulberry, kidney beans, and coffee beans. In kidney beans, phaseolamin has been suggested as the responsible compound that can lead to a reduction in the food intake and body weight of experimental animals [19,22]. Caffeine, mainly by increasing energy expenditure, and, to a lesser extent, chlorogenic acid has anti-obesity activity in coffee and coffee bean extracts [23], whereas, in mulberry leaves and fruits, many bioactive compounds can be responsible for this effect. The most active compounds against obesity seem to be flavonoids and iminosugars [24]; however, an increasing number of studies also highlight specific mulberry polysaccharides as potent in vitro inhibitors of preadipocyte cell proliferation [25]. Moreover, in the present study, the feeding experiment confirmed the glucose-lowering activity observed for mixture 1 in the tolerance tests. The slightly increased plasma glucose level in the HF group significantly decreased in the HF+Mix1 group; however, the plasma insulin level did not differ between the groups (Table 4). Moreover, mixture 1 was able to prevent the increase in plasma non-HDL cholesterol levels caused by the HF diet, which we consider as an additional benefit that results from the mixture ingestion. Taking all of the observed beneficial effects of mixture 1 into consideration, the results of this study have become the basis for performing clinical trials in which the glucose-lowering effect can be verified. 

## 4. Materials and Methods

### 4.1. Preparations

Standardized extracts from white mulberry (*Morus alba* L.), white kidney (*Phaseolus vulgaris* L.), robusta coffee (*Coffea robusta*), bitter melon (*Momordica charantia* L.), and pomelo (*Citrus aurantium* L.) were the main preparations used in this study. White mulberry leaf extract containing 5.12% 1-deoxynojirimycin was purchased from Sino Nutrition (Fujian, China). White kidney bean extract with 4000 units of amylase inhibitory activity per g of the extract was obtained from Monteloeder (Alicante, Spain). Green coffee bean extract containing 60% chlorogenic acids (45% 5-caffeoylquinic acid and 15% 3-caffeoylquinic acid) and 1% caffeine was obtained from Dynadis (Auterive, France). Bitter melon fruit extract was obtained from Hangzhou New Asia International (Hangzhou, China). Pomelo fruit extract containing 30.2% naringenin was obtained from Sino Nutrition (Fujian, China).

l-Arabinose and acarbose were purchased from Sigma-Aldrich, St. Louis, USA (≥99% and ≥95% purity, respectively). Soluble starch was purchased from POCH Joint-Stock Company (≥99% purity, Gliwice, Poland). Sucrose was purchased from Standard Ltd., Poland.

### 4.2. Animals

A total of 160 male Wistar rats were used in this study. All rats were maintained in wired cages under a 12 h light/dark cycle, a controlled temperature of 19 °C to 22 °C and intensive room ventilation (15 times per h). The study was conducted in compliance with European guidelines for the care and use of laboratory animals (EU Directive 2010/63/EU for animal experiments). The animal protocol used in this study was approved by the Institutional Animal Care and Use Committee in Olsztyn, Poland (permission number: 42/22013).

### 4.3. Oral Tolerance Tests

The oral sucrose and starch tolerance tests (SuTT and StTT, respectively) were divided into two stages and were conducted on a total of 130 rats with an average body weight (BW) of 381 ± 16.6 g. In stage 1, single preparations and extracts were given to rats by gavage, whereas in stage 2, mixtures composed of selected preparations were gavaged. Before each stage, rats were starved overnight, but they had free access to tap water. Prior to each test, the water was removed, and the rats were distributed into groups of 10 animals each. Five minutes before a tolerance test, they were gavaged with single portions of water (control group), an aqueous solution of acarbose (positive control; 35 mg/kg BW) or an aqueous solution of the preparations or their mixtures (stage 1 or stage 2, respectively). In stage 1, the following preparations and their doses per kg BW were tested: arabinose (25 mg), mulberry leaf extract (120 mg; MLE group), coffee bean extract (20 mg; CBE group), kidney bean extract (100 mg; KBE group), bitter melon fruit extract (150 mg; BME group), and pomelo fruit extract (100 mg; PFE group). Doses of preparations were calculated on the basis of the body surface area normalization method [26] to prioritize their affordability for use in future clinical trials. Based on the results from stage 1, mixtures of the preparations and their doses per kg BW shown in Table 5 were tested in stage 2. Five minutes after gavage with the preparations or their mixtures, rats intragastrically received 2 g/kg BW sucrose or starch (SuTT or StTT, respectively). A drop of blood was then collected from the tail tip, and the glucose concentration was measured using a glucometer (Accu-Chek Go, Roche Diagnostics, Mannheim, Germany) at the following time intervals: 0, 15, 30, 60, 90, 120, and 180 min. The glycemic response during the SuTT and StTT was evaluated by total area under the blood glucose curve (AUC) using the trapezoidal rule. All solutions in the tests were prepared so that their amounts were 1 mL per 350 g of BW, whereas the control group received water in the same amount.

### 4.4. Feeding Experiment

The feeding experiment was conducted on 30 rats and consisted of a 10 week-long preliminary stage with a regular or high-fat (HF) diet and then a 10 week-long proper stage, in which a standard semipurified casein diet and its HF modifications were applied. At the beginning of the preliminary stage, rats aged 35 days were allocated to the control (10 rats) or the HF groups (20 rats), and they were fed for 10 weeks with the following commercial diets: the control group with a regular chow diet (C 1090-10, Altromin GmbH, Lage, Germany), in which 10% kcal originated from fat, whereas the HF group with a commercial HF diet (C 1090-45, Altromin GmbH), in which 45% kcal originated from fat. After the preliminary stage, 10 rats on the regular chow diet were then switched to a standard, semipurified casein diet, whereas 20 rats on the HF diet were randomly distributed into 2 groups of 10 animals each and were then fed with an HF modification of the semipurified diet supplemented or not with mixture 1 (group HF+Mix1 or HF, respectively). Details on the semi-purified diets are shown in Table 6. Mixture 1 was chosen for the feeding experiment, taking into account the results of the oral tolerance tests. The dietary amount of mixture 1 was adjusted during the 10 week experimental feeding to reflect its dosage used in the oral tolerance tests. To meet the dosage per kg BW, the composition of the diet fed to the HF+Mix1 group was changed every 2 weeks.

### 4.5. Body Composition Analysis

At the beginning and end of the feeding experiment, the body composition of rats (lean and fat mass) was determined by time-domain nuclear magnetic resonance using a Minispec LF 90II analyzer (Bruker, Karlsruhe, Germany). The Minispec analyzer transmits various radiofrequency pulse sequences into soft tissues to reorient the nuclear magnetic spins of the hydrogen atoms and then detects radio frequency signals generated by the hydrogen spins from these tissues. The contrast in relaxation times of the hydrogen spins found between adipose tissue and water-rich tissues are used to estimate fat and lean body mass.

### 4.6. Biochemical Analysis of Blood

After 10 weeks of the proper stage of feeding, rats were anesthetized with an intraperitoneal injection of xylazine and ketamine mixed in physiological salt (10 mg and 100 mg/kg BW, respectively). Animals were then weighed, and their abdomens were cut open. Blood samples were collected from the vena cava into heparinized tubes and centrifuged for 10 min at 380× *g* and 4 °C; the obtained plasma was then stored at –70 °C until analysis.

The plasma concentrations of cholesterol (total and its HDL fraction), triglycerides and glucose were determined using an automatic biochemical analyzer (Pentra C200, Horiba Ltd., Kyoto, Japan). The plasma non-HDL cholesterol concentration was then calculated as the difference between the total and HDL cholesterol concentrations. The plasma insulin concentration was determined using validated rat enzyme immunoassay kits manufactured by Demeditec Diagnostics GmbH (Kiel, Germany).

### 4.7. Statistical Analysis

Statistica software (StatSoft Corp., Krakow, Poland) was used to determine whether the variables differed among the treatment groups. The statistical analysis was performed using a one-way analysis of variance (ANOVA) and Duncan’s multiple range post hoc test. If the variance was unequal, the Kruskal–Wallis ANOVA by ranks was used followed by Dunn’s post hoc test. The results are presented as the mean and the standard error of the mean (SEM). Differences were considered significant at *p* ≤ 0.05.

## 5. Conclusions

To conclude, a mixture consisting of mulberry leaf, kidney bean, and coffee bean extracts with mass percentages of 50, 42, and 8, respectively, was found to be the most effective inhibitor of postprandial glycemia in rats. The overall effective dosage of the mixture was 240 mg per kg of rat BW, and according to the body surface area normalization method, this represents approximately 2.8 g for an adult weighing 70 kg (Reagan-Shaw et al., 2008), which is a dose feasible for use in clinical trials. The glucose-lowering effect of the mixture and its effective dosage were confirmed in the long-term feeding experiment on rats fed the HF diet. Dietary supplementation with the mixture was also able to attenuate other disorders induced by the HF diet, including the increase in body weight, body fat, and blood cholesterol. Moreover, further research on the effects of the mixture on gastrointestinal tract are needed since it is known that enzyme inhibitors can negatively affect its functions.

## Figures and Tables

**Table 1:** The area under the blood glucose curve for the oral sucrose and starch tolerance tests in rats gavaged with acarbose, arabinose, or extracts.

Group	SuTT–AUC (mmol/L × 180 min)	StTT–AUC (mmol/L × 180 min)
Control	1281 ± 20.0 ^a^	1173 ± 16.4 ^a,b^
Acarbose	994 ± 21.7 ^d^	994 ± 21.8 ^d^
Arabinose	1277 ± 18.1 ^a^	1091 ± 13.7 ^c^
MLE	1175 ± 13.3 ^c^	1101 ± 12.2 ^c^
CBE	1220 ± 10.9 ^b,c^	1140 ± 15.8 ^a,b,c^
KBE	1202 ± 16.2 ^b,c^	1185 ± 17.8 ^a^
PFE	1217 ± 19.8 ^b,c^	1133 ± 15.4 ^b,c^
BME	1244 ± 15.9 ^a,b^	1138 ± 17.5 ^a,b,c^
ANOVA *p*-value	<0.001	<0.001

Values are expressed as the mean ± SEM (*n* = 12). AUC, area under the blood glucose curve; BW, body weight; SuTT, sucrose tolerance test; StTT, starch tolerance test; MLE, mulberry leaf extract (120 mg/kg BW); CBE, coffee bean extract (20 mg/kg BW); KBE, kidney bean extract (100 mg/kg BW); BME, bitter melon fruit extract (150 mg/kg BW); PFE, pomelo fruit extract (100 mg/kg BW). ^a, b, c, d^ Mean values within a column with differing superscript letters are significantly different at *p* < 0.05 in a *post hoc* test.

**Table 2:** The area under the blood glucose curve for the oral sucrose and starch tolerance tests in rats gavaged with acarbose or composed mixtures.

Group	SuTT–AUC (mmol/L × 180 min)	StTT–AUC (mmol/L × 180 min)
Control	1351 ± 11.7 ^a^	1174 ± 16.5 ^a^
Acarbose	1097 ± 14.2 ^c^	966 ± 14.7 ^c^
Mix1	1181 ± 16.8 ^b^	1073 ± 12.4 ^b^
Mix2	1177 ± 12.4 ^b^	1084 ± 16.1 ^b^
Mix3	1202 ± 17.2 ^b^	1063 ± 17.1 ^b^
ANOVA *p*-value	<0.001	<0.001

Values are expressed as the mean ± SEM (*n* = 12). AUC, area under the blood glucose curve; BW, body weight; SuTT, sucrose tolerance test; StTT, starch tolerance test. Mean values within a column with differing superscript letters (^a, b, c^) are significantly different at *p* < 0.05 in a *post hoc* test. Composition and doses (mg/kg BW): Mix1—mulberry leaf extract (120 mg) + kidney bean extract (100 mg) + coffee bean extract (20 mg); Mix2—mulberry leaf extract (120 mg) + coffee bean extract (20 mg) + arabinose (25 mg); Mix3—mulberry leaf extract (120 mg) + kidney bean extract (100 mg) + coffee bean extract (20 mg) + arabinose (25 mg).

**Table 3:** Effects of mixture 1 on the diet intake, body weight, and body composition of rats fed a high-fat diet.

Index	Group			ANOVA *p*-Value
	Control	HF	HF+Mix1	
Initial BW (g)	348 ± 10.6	364 ± 11.2	366 ± 6.4	NS
Diet intake (g/10 weeks)	1270 ± 22.7 ^a^	1091 ± 18.5 ^b^	1050 ± 16.4 ^b^	<0.001
Final BW (g)	427 ± 10.9 ^b^	484 ± 13.1 ^a^	469 ± 4.99 ^a^	0.001
BW gain (g)	78.9 ± 4.14 ^c^	121 ± 5.52 ^a^	103 ± 6.31 ^b^	<0.001
Body composition				
Initial fat (% BW)	34.9 ± 0.612	36.0 ± 0.646	35.2 ± 0.504	NS
Initial lean (% BW)	36.1 ± 1.007 ^a^	33.8 ± 0.960 ^b^	33.1 ± 0.508 ^b^	<0.05
Final fat (% BW)	38.6 ± 0.699 ^b^	42.9 ± 0.757 ^a^	41.4 ± 0.614 ^a^	<0.001
Final lean (% BW)	29.6 ± 0.948 ^a^	21.4 ± 1.190 ^b^	23.0 ± 0.843 ^b^	<0.001
Fat gain (g)	43.3 ± 3.18 ^c^	76.9 ± 4.00 ^a^	65.5 ± 4.01 ^b^	<0.001
Lean gain (g)	1.24 ± 3.24 ^a^	−19.5 ± 4.49 ^b^	−13.1 ± 3.04 ^b^	0.001

Values are expressed as the mean ± SEM (*n* = 12). BW, body weight; HF, high-fat; HF+Mix1, high-fat supplemented with mulberry leaf extract (120 mg/kg BW), kidney bean extract (100 mg/kg BW) and coffee bean extract (20 mg/kg BW). Mean values within a row with differing superscript letters (^a, b, c^) are significantly different at *p* < 0.05 in a *post hoc* test.

**Table 4:** Effects of mixture 1 on the plasma markers of glucose and lipid metabolism in rats fed a high-fat diet.

Index	Group			ANOVA *p*-Value
	Control	HF	HF+Mix1	
**Glucose metabolism**				
Glucose (mmol/L)	10.0 ± 0.543 ^a,b^	10.9 ± 0.416 ^a^	9.09 ± 0.625 ^b^	<0.05
Insulin (pmol/L)	256 ± 35.5	318 ± 26.2	304 ± 48.7	NS
Lipid profile				
Cholesterol (mmol/L)	2.30 ± 0.124	2.41 ± 0.121	2.10 ± 0.076	NS
HDL cholesterol (mmol/L)	1.82 ± 0.096	1.79 ± 0.100	1.63 ± 0.085	NS
Non-HDL cholesterol (mmol/L)	0.480 ± 0.030 ^b^	0.616 ± 0.051 ^a^	0.478 ± 0.030 ^b^	<0.05
Triglycerides (mmol/L)	1.70 ± 0.145	1.74 ± 0.094	1.41 ± 0.135	NS

Values are expressed as the mean ± SEM (*n* = 12). BW, body weight; HF, high-fat; HF+Mix1, high-fat supplemented with mulberry leaf extract (120 mg/kg BW), kidney bean extract (100 mg/kg BW) and coffee bean extract (20 mg/kg BW). Mean values within a row with differing superscript letters (^a, b, c^) are significantly different at *p* < 0.05 in a *post hoc* test.

**Table 5:** Recipes of mixtures administered to rats via gavage.

Mixture	Extract/Preparation	Dose (mg/kg BW)	Mass Percentage
Mix1	Mulberry leaf extract	120	50
	Kidney bean extract	100	42
	Coffee bean extract	20	8
Mix2	Mulberry leaf extract	120	73
	Green coffee bean extract	20	12
	Arabinose	25	15
Mix3	Mulberry leaf extract	120	45
	Kidney bean extract	100	38
	Green coffee bean extract	20	8
	Arabinose	25	9

BW, body weight.

**Table 6:** Composition of the diets fed to rats for 10 weeks at the proper stage of the experiment.

Ingredient	Group		
	C	HF	HF+Mix1
Casein	22.5	22.5	22.5
DL-methionine	0.2	0.2	0.2
Rapeseed oil (canola)	5	-	-
Lard	-	24	24
Cellulose	5	5	5
Corn starch	62.6	43.6	42.984–42.808 *
MLE	-	-	0.308–0.396 *
KBE	-	-	0.257–0.330 *
CBE	-	-	0.051–0.066 *
Vitamin mix ^1^	1	1	1
Mineral mix ^1^	3.5	3.5	3.5
Choline chloride	0.2	0.2	0.2

BW, body weight; MLE, mulberry leaf extract; KBE, kidney bean extract; CBE, coffee bean extract. ^1^ Recommended for the AIN-93 diet [27]. * The composition of the diet was changed every 2 weeks of feeding to adjust the extract dosages to the increases in rats’ BW (in mg/kg BW: mulberry leaf extract, 120; kidney bean extract, 100; coffee bean extract, 20).
